# Proteomic Profiling of Chemotherapy Responses in FOLFOX-Resistant Colorectal Cancer Cells

**DOI:** 10.3390/ijms24129899

**Published:** 2023-06-08

**Authors:** Shing-Yau Tam, Md Zahirul Islam Khan, Ju-Yu Chen, Jerica Hiu-Yui Yip, Hong-Yiu Yan, Tsz-Yan Tam, Helen Ka-Wai Law

**Affiliations:** Department of Health Technology and Informatics, Faculty of Health and Social Sciences, The Hong Kong Polytechnic University, Kowloon, Hong Kong

**Keywords:** chemoresistance, colorectal cancer, cytoskeleton, FOLFOX, proteomic profiling, ribosome

## Abstract

Chemoresistance mechanisms of colorectal cancer remain largely elusive. We aim to compare the difference of chemotherapy responses between FOLFOX-resistant and wild-type colorectal cancer cells by proteomic profiling to suggest novel treatment targets. FOLFOX-resistant colorectal cancer cells DLD1-R and HCT116-R were developed by chronic exposure to progressive FOLFOX doses. Proteomic profiling of FOLFOX-resistant and wild-type cells under FOLFOX exposure were conducted by mass-spectrometry-based protein-analysis technology. Verification of selected KEGG pathways was conducted by Western blot. DLD1-R had significantly higher FOLFOX-chemoresistance (10.81 times) than its wild-type counterpart. A total of 309 and 90 differentially expressed proteins were identified in DLD1-R and HCT116-R, respectively. In terms of gene ontology molecular function, RNA binding and cadherin binding ranked first for DLD1 and HCT116 groups, respectively. For gene set enrichment analysis, ribosome pathway and DNA replication were significantly up-regulated and down-regulated in DLD1-R, respectively. The most significantly up-regulated pathway in HCT116-R was regulation of the actin cytoskeleton. Up-regulations in the ribosome pathway (DLD1-R) and actin cytoskeleton (HCT116-R) were verified by Western blot. There were several significantly altered signaling pathways in FOLFOX-resistant colorectal cancer cells under FOLFOX with notable up-regulations in the ribosomal process and actin cytoskeleton.

## 1. Introduction

Colorectal cancer is the third most common cancer diagnosed worldwide with an estimated incidence of 1.93 million in 2020 [1]. It is also the second leading cause of cancer-related mortality with 0.94 million cases in 2020 [1]. Surgery, chemotherapy and radiotherapy have been developed as the major treatment modalities for colorectal cancer. Although the control of disease is possible through the developed modalities, there is still room for improvement of overall survival. The survival of colorectal cancer patients is mainly affected by metachronous metastasis. The 5-year overall survival for Stage IV metastatic cases is 10.5–28.1%, which is significantly lower than Stage III (73.2%) [2,3].

Chemotherapy has been established as standard of care for patients with Stage II or above colorectal cancer for survival benefits [4]. A fluorinated pyrimidine that acts by inhibition of thymidylate synthetase, 5-fluorouracil (5-FU), has been long established as the first-line therapy along with leucovorin (LEU), which increases the efficacy of 5-FU [4]. These drugs can combine with other chemotherapy drugs such as oxaliplatin (OXA) or irinotecan as the combined treatment regimens FOLFOX and FOLFIRI, respectively [4]. OXA is an alkylating agent for inducing DNA damage, which has been demonstrated to further increase overall survival compared with 5-FU + LEU. However, irinotecan, an inhibitor of topoisomerase I, could not produce major benefits in survival [5]. Previous studies showed that FOLFOX treatment could achieve an overall survival of 2 years in metastatic colorectal cancer cases [6]. Nevertheless, only 30–50% of patients showed an objective response to the combination therapies and, thus, the chemoresistance issue is still a major clinical problem in the treatment of metastatic colorectal cancer [7].

Chemoresistance could be caused by tumor cells themselves and tumor-cell-independent factors [5]. The tumor-cell-independent factors include signals from different tumor microenvironment cell populations, hypoxia and inflammation [5]. The intrinsic chemoresistance of colorectal cancer involves the enhanced cellular efflux of chemotherapy drugs with mutations of various genes (KRAS, BRAF, EGFR) and changes in respective signaling pathways [5]. As chemotherapy is usually employed with advanced or metastatic colorectal cancer stages, various intrinsic and drug-dependent mechanisms are likely to be already developed from tumor microevolution processes. The commonly proposed cause of chemoresistance is the aberrant anti-tumor drug metabolism, transportation or target [8]. Cell-death pathways, carcinogenic signals, compensation feedback-loop signal pathways and the tumor immune microenvironment have also been determined to be important processes for the investigation of chemoresistance [8]. Nevertheless, chemoresistance mechanisms remain largely elusive with the major of related studies restricted to preclinical stages.

In recent years, proteomic profiling by mass spectrometry (MS)-based protein analysis technology has been widely used for unravelling key information in tumorigenesis [9,10]. Proteomics-based technologies allow for the identification of potential biomarkers and protein expression patterns to evaluate tumor prognosis and classification as well as identify potential responders for specific therapies. As the chemoresistance mechanisms for colorectal cancer warrant further studies and threaten overall survival, we aim to compare the difference of chemotherapy responses between FOLFOX-resistant and wild-type colorectal cancer cells for suggesting novel treatment targets.

## 2. Results

### 2.1. Verification of Chemoresistance of Cells

The development of chemoresistance was conducted in 2021–2022 and took around one year. The final concentrations achieved were 0.5 mM 5-FU, 50 μM LEU and 50 μM OXA for DLD1-R and 5 μM 5-FU, 0.5 μM LEU and 0.5 μM OXA for HCT116-R. A total of 36 and 18 passages were performed to develop DLD1-R and HCT116-R.

**Figure 1:**
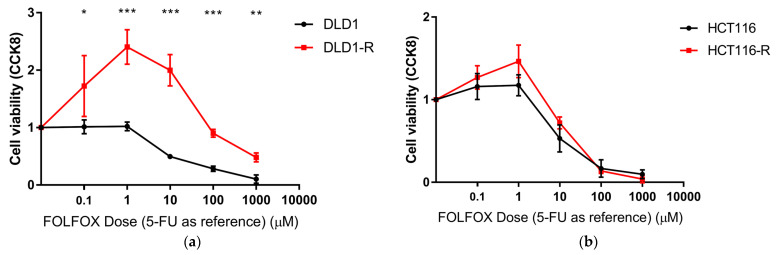
CCK8 test for the verification of chemoresistance. (**a**) DLD1-R had significantly higher chemoresistance than DLD1; (**b**) HCT116-R did not have significantly higher chemoresistance than HCT116. FOLFOX has a 5-FU:LEU:OXA ratio of 10:1:1. Data in mean ± SD. N = 3–4, * *p* < 0.05, ** *p* < 0.01, *** *p* < 0.001.

The confirmation of chemoresistance was conducted by CCK8 testing with a range of FOLFOX concentrations tested. The range was from 100 nM 5-FU, 10 nM LEU and 10 nM OXA to 1 mM 5-FU, 0.1 mM LEU and 0.1 mM OXA. The results showed that the developed DLD1-R was significantly more chemoresistant in various FOLFOX concentrations than DLD1, with 10.81 times higher IC50 (Figure 1a), while the developed HCT116-R was slightly more chemoresistant than HCT116, with 1.61 times higher IC50 and no significant difference in all FOLFOX concentrations (Figure 1b). 

### 2.2. Overview of Proteomics Data

**Figure 2:**
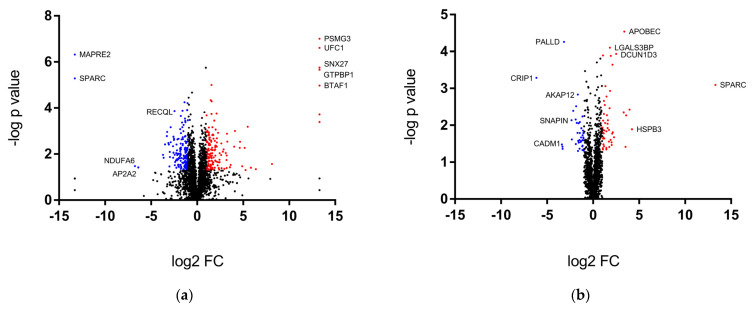

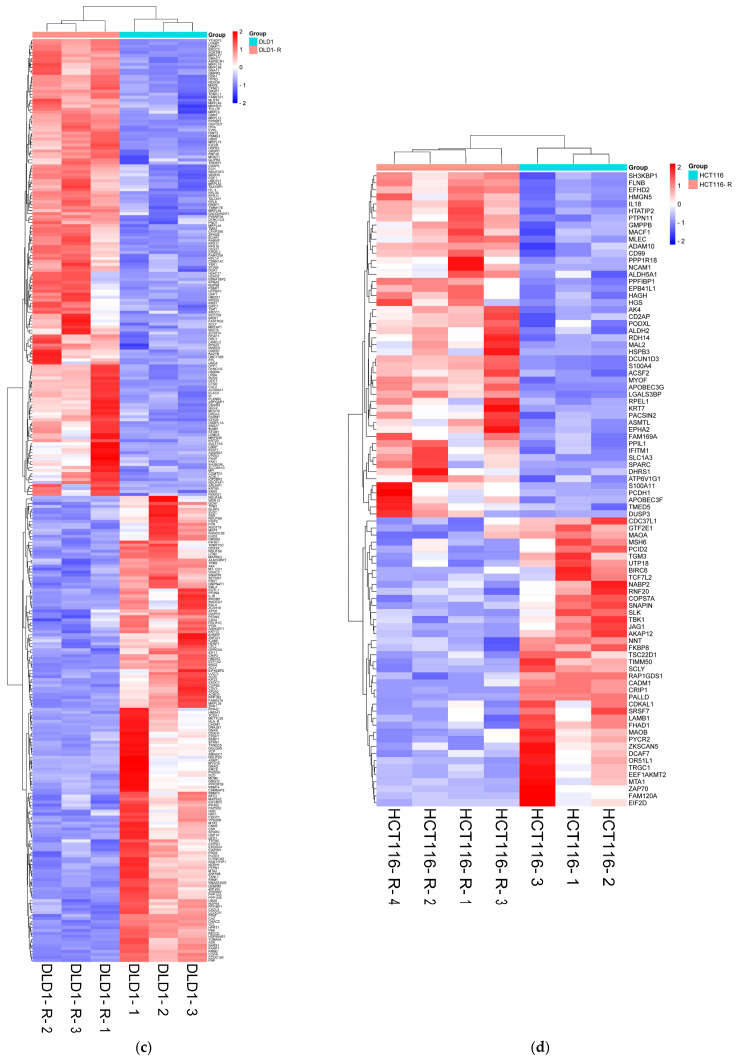
Overall view of proteomics data. The volcano plots of the proteomics data of (**a**) DLD1-R vs. DLD1 and (**b**) HCT116-R vs. HCT116 with red and blue points showing the up-regulated and down-regulated DEPs, respectively. Selected DEPs were marked. The cluster heat maps of DEPs of (**c**) DLD1-R vs. DLD1 and (**d**) HCT116-R vs. HCT116. N = 3–4.

In order to analyze the protein expression changes between chemoresistant and wild-type cells upon FOLFOX treatment, we employed high-throughput MS proteomics analysis. MS analysis identified a total of 2759 (DLD1-R and DLD1) and 2764 (HCT116-R and HCT116) proteins. Among the identified proteins, there were 309 differentially expressed proteins (DEPs) between DLD1-R and DLD1 (−1 ≤ Log2 FC ≥ 1, *p* < 0.05). Of these, 153 and 156 proteins were up- (labeled as red) and down-regulated (labeled as blue), respectively (Figure 2a, Table S1). On the other hand, 90 proteins were DEPs between HCT116-R and HCT116 (−1 ≤ Log2 FC ≥ 1, *p* < 0.05), of which 49 and 41 proteins were up-regulated (labeled as red) and down-regulated (labeled as blue), respectively (Figure 2b, Table S2). The overall expression patterns among groups were displayed in a heat map (Figure 2c,d).

### 2.3. Gene Ontology Analysis of Significantly Enriched Proteins

**Figure 3:**
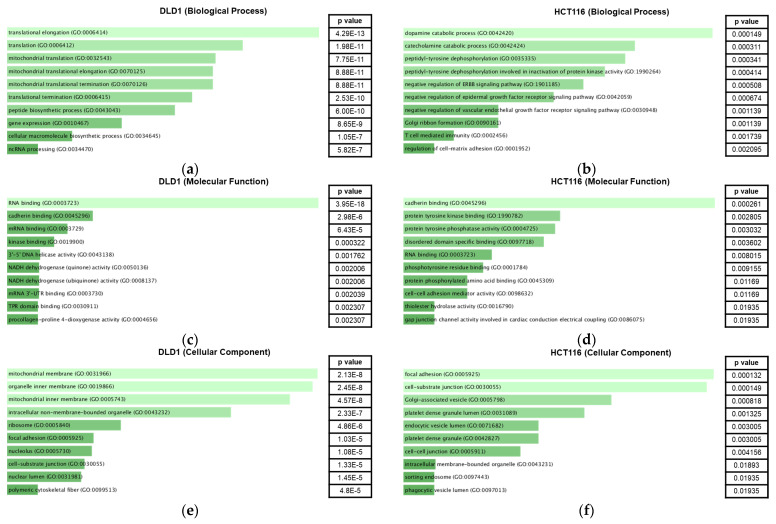
GO analysis of DEPs. GO analysis of biological process, molecular function and cellular component were conducted for the DEPs in DLD1 (**a**,**c**,**e**) and HCT116 (**b**,**d**,**f**) groups. The graphs show the top 10 GO terms according to *p* value with the more significant terms in lighter color. N = 3–4.

We employed Enrichr [11,12], a functional enrichment analysis database, to classify the genes according to their respective gene ontology (GO) terms (Figure 3). G:Profiler was employed to convert DEPs to their corresponding gene identifications. In the GO biological process, translational elongation ranked first (adj *p* value < 0.001) in terms of *p* value for the DLD1 group (Figure 3a) while regulation of cell migration ranked first (adj *p* value < 0.05) for the HCT116 group (Figure 3b). For the GO molecular function, RNA binding (adj *p* value < 0.001) and cadherin binding (adj *p* value < 0.001) ranked first for DLD1 and HCT116 groups, respectively (Figure 3c,d). Cadherin binding ranked second (adj *p* value < 0.001) for the DLD1 group. For the GO cellular component, mitochondrial membrane (adj *p* value < 0.001) and focal adhesion (adj *p* value < 0.05) ranked first for DLD1 and HCT116 groups, respectively (Figure 3e,f).

### 2.4. Gene Set Enrichment Analysis of the Proteomic Data

**Figure 4:**
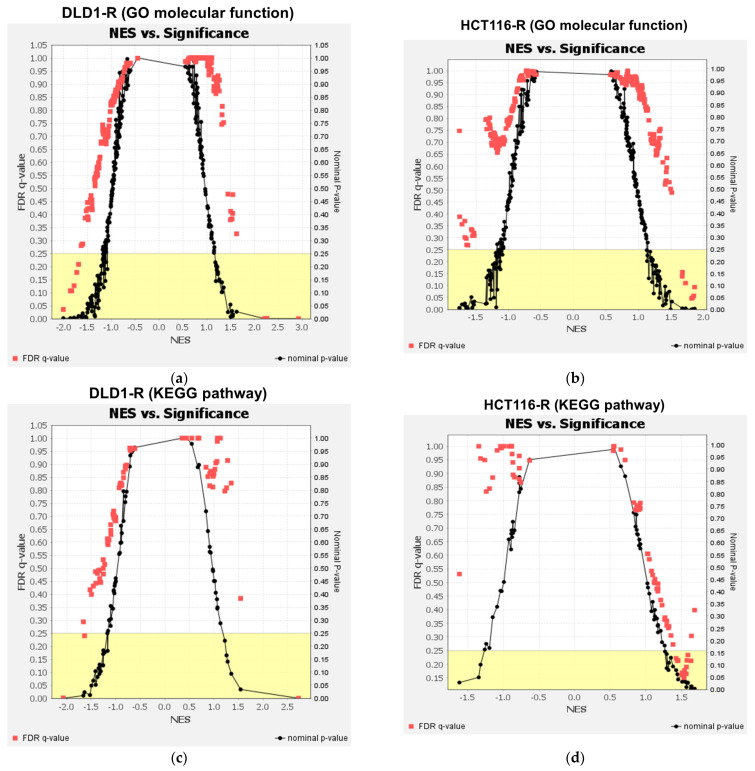
Overview of GSEA analysis. GSEA analysis of GO molecular function for DLD1 (**a**) and HCT116 (**b**) groups showed 9 and 7 processes were significantly dysregulated. For the KEGG pathway, 3 and 13 pathways were significantly dysregulated in DLD1-R (**c**) and HCT116-R (**d**), respectively. N = 3–4.

**Figure 5:**
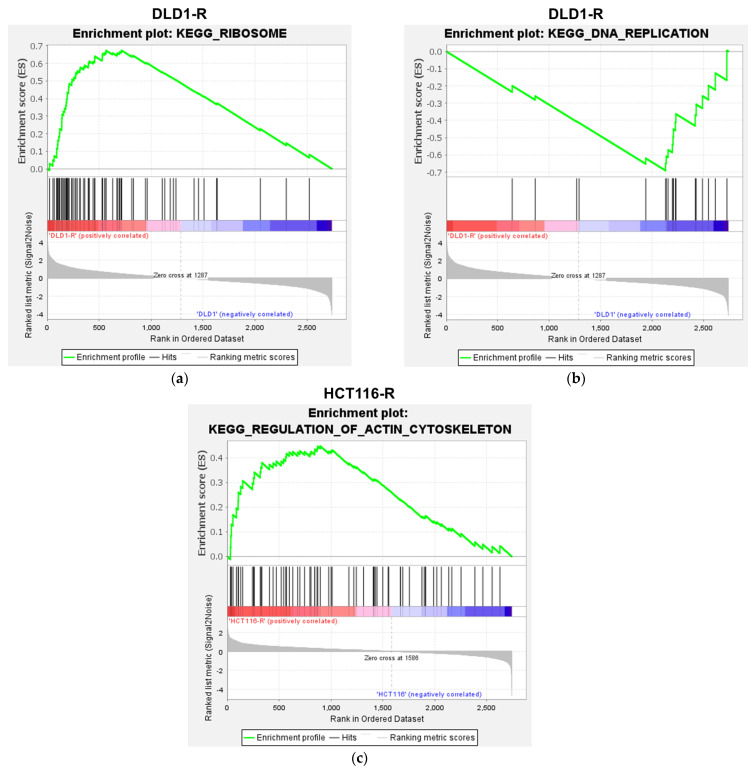
Enrichment plots of selected KEGG pathways in GSEA analysis. For DLD1-R, significant up-regulation of ribosome (**a**) and down-regulation of DNA replication (**b**) were found. For HCT116-R, regulation of actin cytoskeleton was significantly up-regulated (**c**). N = 3–4.

**Figure A1:**
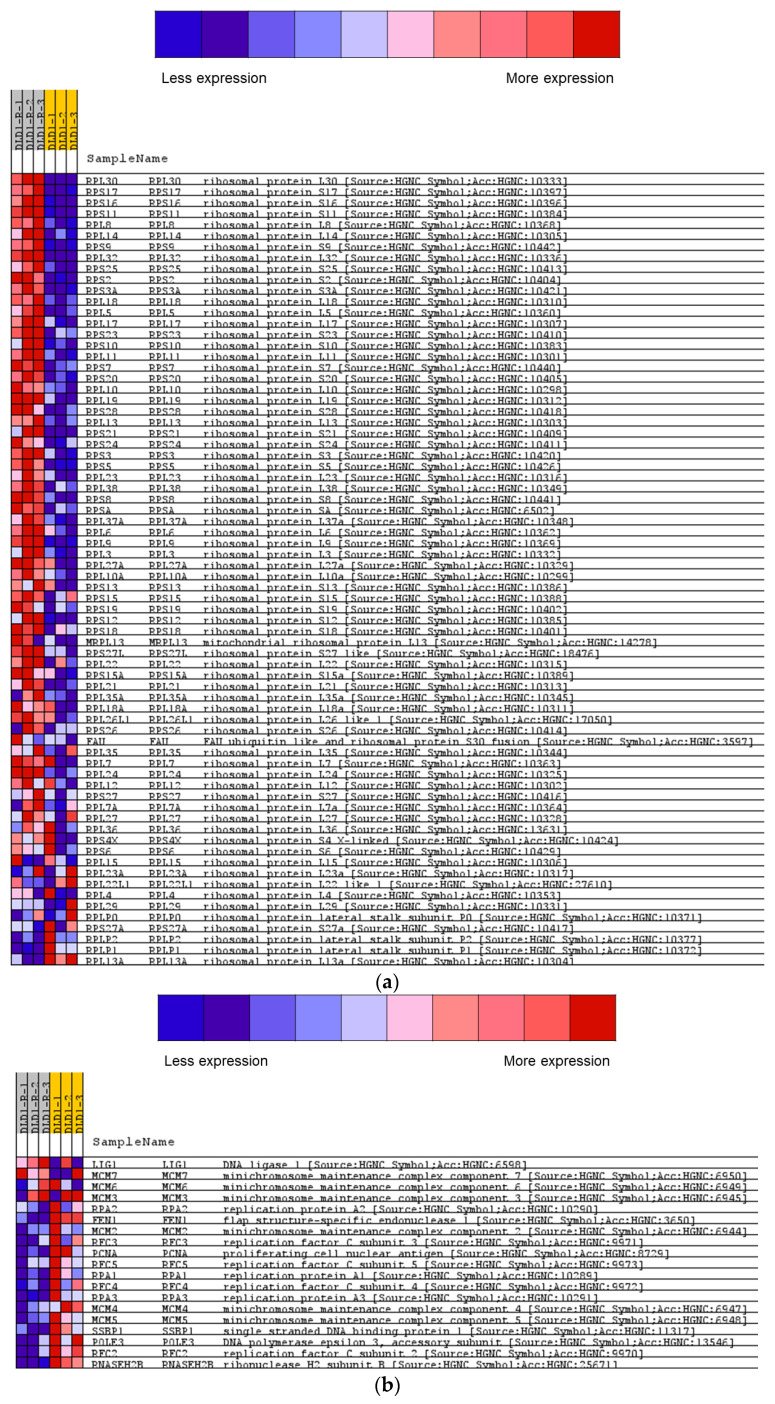

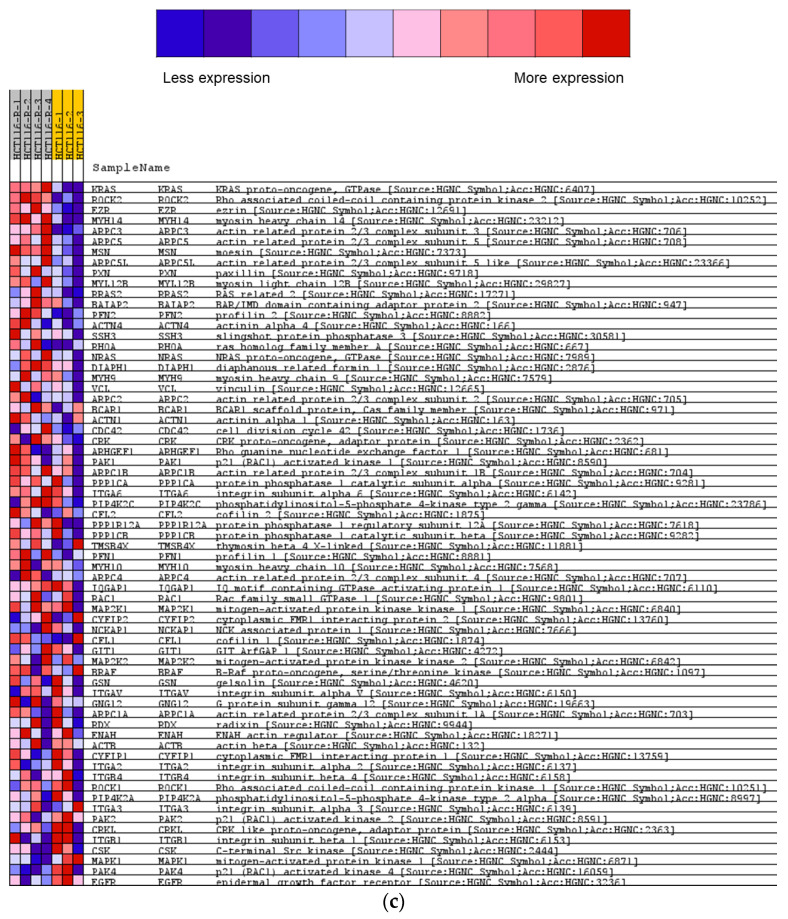
Heat maps of selected KEGG pathways in GSEA analysis. For DLD1-R, significant up-regulation of ribosome (**a**) and down-regulation of DNA replication (**b**) were found. While for HCT116-R, regulation of actin cytoskeleton (**c**) was significantly up-regulated in HCT116-R. N = 3–4.

We continued the analysis at the pathway analysis level in gene set enrichment analysis (GSEA) using the GO molecular function and KEGG human pathway for the whole proteomics data. For GO molecular functions, 3 and 6 processes were significantly up-regulated and down-regulated in DLD1-R (Table 1 and Figure 4a), while 7 processes were significantly up-regulated in HCT116-R (Table 1 and Figure 4b). The up-regulated processes in DLD1-R were mostly related to ribosomal activities while the down-regulated processes were related to DNA activities. The up-regulated processes in HCT116-R were related to actin binding and oxidoreductase activities. For the KEGG pathway, 1 and 2 pathways were significantly up-regulated and down-regulated in DLD1-R (Table 2 and Figure 4c) while 10 pathways were significantly up-regulated in HCT116-R (Table 2 and Figure 4d). Similar to the GO molecular functions, the ribosome pathway was up-regulated and DNA replication was down-regulated in DLD1-R (Figure 5a,b and Figure A1a,b, Tables S3 and S4). The most significantly up-regulated pathway in HCT116-R was related to regulation of the actin cytoskeleton (Figure 5c and Figure A1c, Table S5).

### 2.5. Western Blot Verification of Selected Pathways

**Figure 6:**
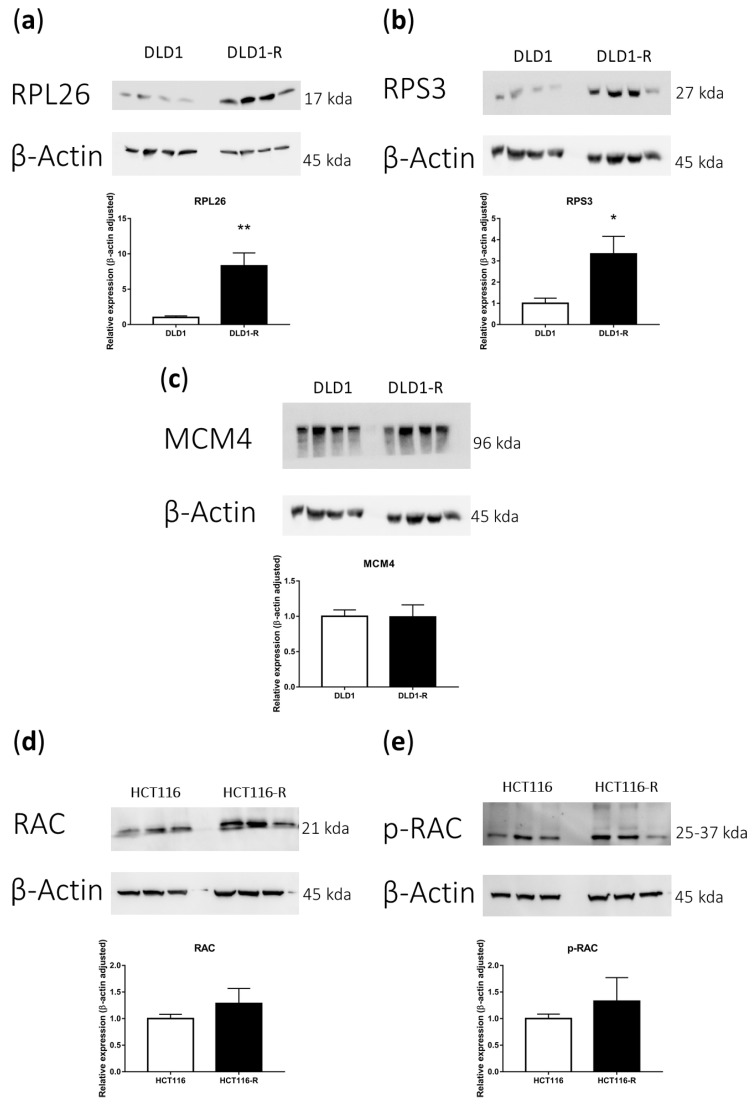
Western blot verification of the proteomics results. Key ribosome markers RPL26 (**a**) and RPS3 (**b**) were significantly up-regulated in DLD1-R. No significant alteration was found for the DNA replication marker MCM4 for DLD1-R (**c**). Key actin-skeleton markers RAC (**d**) and *p*-RAC (**e**) had up-regulation trends in HCT116-R. Data in mean ± SEM, N = 3–4, * *p* < 0.05, ** *p* < 0.01.

For verification of major findings in the proteomics analyses, we performed Western blot for the key markers involved. For DLD1-R, we investigated the key ribosome markers RPL26 and RPS3 to verify the up-regulation of ribosomal processes found in the proteomics analysis. The Western blot results demonstrated that both RPL26 and RPS3 were significantly up-regulated in DLD1-R when compared with DLD1 (Figure 6a,b and Figure S1). For the DNA replication process, we investigated the key marker MCM4, but the results showed that MCM4 was not significantly altered in DLD1-R (Figure 6c and Figure S1). For HCT116-R, we investigated the key regulators of the actin cytoskeleton RAC1 and *p*-RAC1. Results showed that both RAC1 and *p*-RAC1 had up-regulation trends in HCT116-R when compared with HCT116 (Figure 6d,e and Figure S1).

## 3. Discussion

Chemoresistance is an important issue in colorectal cancer treatment, especially for advanced diseases. Although chemotherapy of colorectal cancer has adopted improved multidrug chemotherapy regimens, multidrug chemoresistance is still a common issue found among late-stage diseases and can ultimately cause treatment failure [7]. Various chemotherapy drug resistance mechanisms including tumor-dependent and tumor-independent mechanisms have been proposed, yet most of the drug-resistance mechanisms remain elusive and anti-chemoresistance drug tests still largely remain in the preclinical stage [8]. Therefore, there is a need to discover more underlying resistance mechanisms by a systematical method to understand the major altered processes in the chemoresistant cells.

Proteomics analysis is a commonly used method to discover the protein expression patterns in tumorigenesis processes [9]. This approach could also be applied to the investigation of chemoresistance mechanisms. In addition, colorectal cancer cell lines are usually considered as sufficient for studying drug resistance mechanisms and drug testing [13,14]. In a recent review by Cantor et al. [10], there were some previous proteomics studies on colorectal cancer cell lines for suggesting biomarkers related to resistance of single drugs including 5-FU and dasatinib, but not in multidrug regimens such as FOLFOX. With the immortalized nature of the established colorectal cancer cell lines, the development of FOLFOX-resistant cell lines DLD1-R and HCT116-R by continued FOLFOX exposure with progressively increased dose was possible. By utilizing an in vitro manipulation approach, we could also conduct the subsequent comparisons with wild-type DLD1 and HCT116 [15]. We conducted the proteomics analysis under exposure to FOLFOX to discover the alterations in cellular processes and signaling pathways among chemoresistant colorectal cancer cells. Our study is the first to use this comparison approach according to our knowledge.

During the preliminary analysis of the DEPs, we found 309 and 90 DEPs out of 2759–2764 of the identified proteins for DLD1-R and HCT116-R, respectively. The large number of DEPs showed that there are notable number of proteins altered for the cellular response to chemotherapy drugs after the chemoresistance development process. Further analysis by GO and GSEA have identified up-regulation of the ribosomal process and down-regulation of DNA replication in DLD1-R, and up-regulation of the actin cytoskeleton in HCT116-R. For verification, we conducted Western blotting of the key regulators of the processes involved in the proteomics findings. The results verified our findings in the up-regulation of the ribosomal process in DLD1-R and up-regulation of the actin cytoskeleton in HCT116-R, but failed to verify the down-regulation of DNA replication in DLD1-R.

Up-regulation of ribosomal processes in DLD1-R is the major finding of this study. Ribosome biogenesis has been identified as a major player in cancer tumorigenesis, metastasis and therapy resistance [16]. For example, overexpression of RPL13 has been demonstrated to promote chemoresistance in gastric cancer [17]. For colorectal cancer, ribosomal biogenesis has been regarded as the integrator or final effector of the major altered signaling pathways in tumorigenesis, such as MYC and KRAS [18]. An MYC target gene, PES1, has been shown to have links with chemoresistance in colorectal cancer cells [19]. PES1 transcription in colorectal cancer cells is also found to be mediated by the c-Jun NH2-terminal kinase (JNK) pathway. The same research group also demonstrated that JNK inhibition could down-regulate PES1 and subsequently inhibit ribosome biogenesis and tumorigenesis [20]. KRAS mutations are frequently found in colorectal cancer patients and could be a predictive marker for metastatic disease [18]. The mutant KRAS colorectal cancer cell lines were demonstrated to upregulate the genes involved in ribosome biogenesis [21]. However, there has been no direct evidence linking the overexpression of ribosomal proteins to chemoresistance in colorectal cancer. In our study, we have found a series of ribosomal proteins to be up-regulated in chemoresistant colorectal cancer cells by proteomics analysis (Figure A1a, Table S3). Future studies on the specific roles of the significantly altered ribosomal proteins in chemoresistance may be rewarding for prognosticating chemoresistance of colorectal tumors and discovering novel anti-chemoresistance approaches.

Promotion of the actin cytoskeleton in HCT116-R is the second finding of this study. The actin cytoskeleton has been long regarded for having important roles in epithelial-mesenchymal transition (EMT) and cancer metastases [22]. The small GTPase RAC has been demonstrated to involve in various dynamic cell biological processes including EMT and invasiveness, contributing to colorectal cancer metastasis [23,24,25]. RAC has multiple effector proteins for regulating the actin cytoskeleton [26]. RAC stimulates new actin polymerization for lamellipodium extension at the leading edge, and is required for focal complex assembly [27]. In previous studies, Rac1b overexpression was associated with BRAF mutation and lead to poor prognosis [28]. Rac1b overexpression is also associated with poor outcome of wide-type KRAS/BRAF colorectal cancer treated with FOLFOX/XELOX chemotherapy [29]. Our study has found the promotion of actin binding and the cytoskeleton from proteomics analyses which were confirmed with the up-regulation trends in RAC and *p*-RAC in HCT116-R. However, it is worthy to note that the chemoresistance of HCT116-R is not significantly higher than HCT116. The finding may suggest that prolonged exposure to chemotherapy drugs may induce the development of actin dynamics. More extensive studies in RAC and its homologous form CDC42 are required to obtain the full picture of actin cytoskeleton modeling under chemotherapy.

Despite the fact that our study has identified the significantly altered pathways in chemoresistant colorectal cancer cells under FOLFOX chemotherapy, there are several limitations. First, the development of FOLFOX-resistant HCT116 is difficult and we could not achieve a significantly more FOLFOX-resistant cell line after prolonged exposure to FOLFOX. Thus, the findings in HCT116-R may reflect the effect of prolonged FOLFOX exposure on cellular processes and signaling pathways. As both RAC and *p*-RAC were slightly more expressed in HCT116-R, further prolonged exposure to FOLFOX is suggested in future studies. More FOLFOX-resistant colorectal cancer cell lines may be needed for the study of the full picture of chemoresistance-related signaling pathways and to suggest possible anti-chemoresistance strategies. Second, we have found 3 and 10 significantly altered KEGG pathways by GSEA in DLD1-R and HCT116-R, respectively. As we were limited by resources, we could only verify the most significantly altered ribosomal pathway and actin cytoskeleton pathway for DLD1-R and HCT116-R, respectively. More verifications can be performed on other significantly altered pathways to further verify our findings in the proteomics analysis. Moreover, our research using cell lines could act as the initial discovery stage of chemoresistance mechanisms. Nevertheless, as colorectal cancer has inherent biological and clinical heterogeneity [10], research with the use of clinical colorectal tumor samples is warranted to confirm the alterations of the identified pathways, which may ultimately lead to the suggestion of possible methods to improve treatment outcomes.

## 4. Materials and Methods

### 4.1. Cell Line and Culture Condition

Human colorectal cancer cell lines DLD1 and HCT116 were acquired from Prof. Jun Yu (Faculty of Medicine, The Chinese University of Hong Kong, Hong Kong, China). Both cell lines and the subsequent chemoresistant cells were maintained with 10% fetal bovine serum (FBS) (Excell Bio, Shanghai, China) in Dulbecco’s modified eagle medium (DMEM, Gibco, Waltham, MA, USA). Cell culture was maintained at 37 °C in 5% CO_2_ in 100% humidity.

### 4.2. Development of Chemoresistance

The FOLFOX-resistant colorectal cancer cell lines (DLD1-R and HCT116-R) were developed with a chronic and progressively increased FOLFOX dosage with reference to previous literature [30,31]. Briefly, the starting dosage was 100 nM 5-FU, 10 nM LEU and 10 nM OXA (MedChemExpress LLC, Monmouth Junction, NJ, USA). The cells were cultured for 7 days; then, the surviving cells were subcultured in the same FOLFOX concentration for 7 days. The subculture was repeated for the same FOLFOX concentration with a total of 3 rounds and 21 days. Then, the surviving cells were subcultured at 2–2.5 times higher FOLFOX concentration. The surviving cells were allowed to recover without FOLFOX if there were too few cells for passage. The subculture procedures and increment of FOLFOX concentration were repeated until the cells were incapable of growth under FOLFOX.

The development of chemoresistance were confirmed by a Cell Counting Kit-8 (CCK-8) (MedChemExpress LLC) test. Briefly, 5000 cells were cultured in 96-well plates with DMEM. The cells were allowed to settle overnight. Then, different concentrations of FOLFOX from 1 mM 5-FU, 100 μM LEU and 100 μM OXA to 100 nM 5-FU, 10 nM LEU and 10 nM OXA were added to the wells. The cell viability was tested by CCK-8 after 7 days of FOLFOX incubation according to manufacturer’s instructions.

### 4.3. MS Sample Preparation for Proteomics Analysis

Both chemoresistant (DLD1-R and HCT116-R) and wild-type (DLD1 and HCT116) cells were cultured for the preparation of samples. Five million cells were cultured in DMEM and allowed overnight settling. Afterwards, the medium was changed to FOLFOX-containing medium (20 μM 5-FU, 2 μM LEU and 2 μM OXA) to simulate FOLFOX treatment. Cell pellets were collected after 3 days of culturing and stored in −70 °C. All conditions were conducted with at least 3 replicates.

MS samples were prepared using EasyPep MS Sample Prep Kit (Thermo Scientific, Waltham, MA, USA) according to the manufacturer’s protocol. Briefly, 100 μL of lysis buffer and 1 μL of universal nuclease were added to the cell pellet. After reducing sample viscosity by pipetting up and down, the protein concentrations were measured using Pierce BCA Protein Assay Kit (Thermo Scientific, Waltham, MA, USA). Then, 50 µg of proteins were obtained and the final volume adjusted to 100 µL with lysis solution. Afterwards, reduction and alkylation were performed by using reduction solution and alkylation solution. To block the reduction and alkylation, 10 min of heat incubation was performed. For digestion, reconstituted sequencing-grade trypsin + Lys-C enzyme mixture solution (Thermo Scientific, Waltham, MA, USA) was added to the preparation and incubated at 37 °C overnight, and digestion was stopped by adding digestion stop solution (Thermo Scientific, Waltham, MA, USA). Thereafter, the peptide solutions were desalted and cleaned by using a series of washes in a C18 peptide clean-up column. Lastly, the cleaned peptides were collected by 70% acetonitrile in water with 0.1% trifluoroacetic acid elution and were dried using a Refrigerated CentriVap Centrifugal Concentrator (Labconco Corporation, Kansas City, MO, USA).

### 4.4. LC-MS/MS Analysis

The process is the same as our previously published method [32]. LC-MS/MS acquisition was conducted on an Orbitrap Fusion Lumos Mass Spectrometer (Thermo Scientific, Waltham, MA, USA) in the University Research Facility in Chemical and Environmental Analysis, Hong Kong Polytechnic University. For the fractioning, a Dionex Ultimate 3000 RSLCnano System decorated with Acclaim PepMap C18 analytical columns (Thermo Scientific, Waltham, MA, USA) and Trap Column Cartridges Holders with nanoViper Fittings (Thermo Scientific, Waltham, MA, USA) was used. Gradient settings are as follows: solvent A, 0.1% formic acid in Milli-Q; solvent B, 0.1% formic acid in LC/MS-grade acetonitrile (ACN). Gradient started at 2% B at 0–5 min, 6% B at 5–7 min, 20% B at 7–82 min, 30% B at 82–90 min, 90% B at 90–100 min, hold until 105 min and then re-equilibrate with 2% B at 105–115 min. Flow rate of the whole gradient was set as 300 nl/min. Samples with a volume of 1 µL were injected for fractioning and the collected peptide data from the described experiments were analyzed by an Orbitrap Fusion Lumos Mass Spectrometer using a data-dependent acquisition (DDA) strategy in positive ion mode with scan range of 400–1500 m/z, resolution of 60,000 and standard automatic gain control (AGC) target. Charge-state screening was adopted with 2–7 included. The intensity threshold was set at 10,000, whereas dynamic exclusion duration was set as 40 s after one time of acquisition. Selected precursors were fragmented using high-energy collision dissociation (HCD) with normalized collision energy set as 30%. MS/MS was acquired using an Orbitrap as mass analyzer with mass resolution 7500 and standard AGC target. The mass spectrometry proteomics data have been deposited to the ProteomeXchange Consortium via the PRIDE [33] partner repository with the dataset identifier PXD042136.

### 4.5. MS Data Analysis

MS and MS/MS peptides spectra were analyzed by Progenesis QI for Proteomics (Version 4.2, Nonlinear Dynamics, Newcastle, UK) software and Mascot Server 2.5 (Matrix Science, London, UK) using Homo sapiens’ reviewed proteome on Uniprot as the searching database (Sequence of Homo sapiens, Release date: 19 August 2021). The parameters for the Mascot database search were as follows: precursor tolerance level, 10 ppm; fragment tolerance level, 0.05 Da; maximum mass cleavage, 2; maximum number of ^13^C, 1; peptides charge, (2+ to 4+); fixed modification, carbomidomethylation on cysteine residues; variable modification, oxidation on methylation residues. The 1% False Discovery Rate (FDR) was applied to identify the peptides from complex mixtures. Proteins are compared with the Top-N protocol in Progenesis QI for Proteomics with N = 3–4. A fold change (FC) more than 2, *p* < 0.05 (one-way ANOVA) and replicated proteins in all samples were counted as DEPs between the chemoresistant and wild-type cells to complete the analysis.

Graphpad Prism Version 7 was used to generate the volcano plot data from raw proteomics. Heat map data for the DEPs was produced by SRPlot (https://www.bioinformatics.com.cn/plot_basic_cluster_heatmap_plot_024_en (accessed on 4 June 2023). Hierarchical clustering using the average cluster method and Euclidean distance method was employed for the generation of cluster heat maps. Protein (Uniprot) to gene (Entrezgene) conversion was conducted by g:Profiler (https://biit.cs.ut.ee/gprofiler/convert (accessed on 4 June 2023)). GO and pathway analyses of significantly enriched protein of each group (*p* < 0.05, −1.0 ≤ Log2FC ≥ 1.0) were generated by a web-based tool called Enrichr (https://amp.pharm.mssm.edu/Enrichr/ (accessed on 4 June 2023)) [11,12] with default settings. Gene set enrichment analysis (GSEA, version 4.1.0) software (https://www.gsea-msigdb.org/gsea/index.jsp (accessed on 4 June 2023)) was employed to identify the enriched KEGG pathways and molecular functions in the comparison between chemoresistant and wild-type cells upon FOLFOX treatment. Pathways or functions with −1 < Normalized enrichment score (NES) > 1, normalized *p* value < 0.05 and false discovery rate (FDR) < 0.25 were considered as significantly enriched processes. All bioinformatics analysis was performed between February and March, 2023.

### 4.6. Western Blotting

Western blotting was performed using the standard protocol as previously published [34,35]. One million cells were cultured in a T25 flask with DMEM. After overnight settling, the medium was switched to FOLFOX-containing medium with the same FOLFOX concentration used in the proteomics experiment. Whole-cell proteins were collected by 2x loading dye after 3 days of incubation. The proteins were denatured by 5 min of 95 °C heat incubation. Similar amounts of protein were loaded and run on SDS-PAGE. Proteins were then transferred onto Immun-Blot PVDF Membrane (Bio-Rad Laboratories, Inc., Hercules, CA, USA), followed by 30 min blocking in 5% bovine serum albumin (BSA) (Excell Bio) in Tris-buffer saline with a supplement of 0.1% Tween 20 (TBST). Then, the blocked membrane was incubated overnight with primary antibodies β-Actin (#8457, Cell Signaling Technology, Inc., (CST, Danvers, MA, USA)), MCM4 (D3H6N) (#12973, CST), RAC1 (#PA1-091, Invitrogen, Waltham, MA, USA), phosphor-RAC1 (Ser71) (#PA5-104640, Invitrogen), RPL26 (D8F6) (#5400, CST) and RPS3 (D50G7) (#9538, CST) at 4 °C and 1:1000 concentration in TBST with 5% BSA. The membrane was then incubated by the secondary anti-rabbit IgG, horseradish peroxide (HRP)-linked (#7074, CST) antibodies for 1 h at room temperature and 1:6000 concentration in TBST with 5% BSA. SuperSignal West Pico PLUS Chemiluminescent Substrate (ECL) (Thermo Scientific) was added to the membrane according to the manufacturer’s instructions to visualize protein bands in an iBright Imaging System (Invitrogen). The relative protein expressions were quantified using ImageJ software Version 1.54c (NIH) with the internal control of β-actin. All conditions were conducted with at least 3 replicates. Statistical analysis was conducted by Statistical Product and Service Solutions (SPSS, IBM, Armonk, NY, USA) and *p* < 0.05 was considered as statistically significant.

## 5. Conclusions

This study has revealed several significantly altered signaling pathways in FOLFOX-chemoresistant colorectal cancer cells under FOLFOX chemotherapy with notable up-regulations in the ribosomal process and actin cytoskeleton. This study provides insight into the mechanisms of chemoresistant colorectal cancer cells by the systematical proteomics analysis approach. Further research is warranted in exploiting these pathways to improve the treatment efficacy of chemoresistant colorectal tumors and to translate these findings to the clinical level.

## Figures and Tables

**Table 1:** List of significantly dysregulated processes in the GO molecular function in GSEA.

Cell Type	GO Molecular Function	Up-Regulated or Down-Regulated	NES	*p* Value
DLD1-R	Structural constituent of ribosome	Up-regulated	2.93	<0.001
	Structural molecule activity	Up-regulated	2.26	<0.001
	rRNA binding	Up-regulated	2.22	<0.001
	ATP-dependent activity acting on DNA	Down-regulated	−1.69	0.01
	Electron transfer activity	Down-regulated	−1.72	<0.001
	Histone deacetylase binding	Down-regulated	−1.78	0.004
	Glycosyltransferase activity	Down-regulated	−1.83	<0.001
	Disordered domain-specific binding	Down-regulated	−1.86	0.002
	DNA helicase activity	Down-regulated	−2.01	0.002
HCT116-R	Oxidoreductase activity acting on the aldehyde or oxo group of donors	Up-regulated	1.86	0.003
	Actin binding	Up-regulated	1.85	<0.001
	Oxidoreductase activity acting on the aldehyde or oxo group of donors NAD or NADP as acceptor	Up-regulated	1.83	0.002
	Actin filament binding	Up-regulated	1.81	<0.001
	Hydrolase activity acting on carbon nitrogen but not peptide bonds	Up-regulated	1.72	0.005
	Calcium ion binding	Up-regulated	1.67	0.001
	Ubiquitin-like protein binding	Up-regulated	1.67	0.005

**Table 2:** List of significantly dysregulated processes in the KEGG pathway in GSEA.

Cell Type	GO Molecular Function	Up-Regulated or Down-Regulated	NES	*p* Value
DLD1-R	Ribosome	Up-regulated	2.72	<0.001
	Dilated cardiomyopathy	Down-regulated	−1.64	0.023
	DNA replication	Down-regulated	−2.07	<0.001
HCT116-R	Regulation of actin cytoskeleton	Up-regulated	1.63	0.004
	Valine leucine and isoleucine degradation	Up-regulated	1.59	0.028
	Leukocyte transendothelial migration	Up-regulated	1.58	0.027
	Endocytosis	Up-regulated	1.57	0.009
	Propanoate metabolism	Up-regulated	1.57	0.029
	Vasopressin-regulated water reabsorption	Up-regulated	1.54	0.042
	Tight junction	Up-regulated	1.54	0.035
	Pyruvate metabolism	Up-regulated	1.54	0.032
	Lysine degradation	Up-regulated	1.51	0.031
	Fc-gamma-R-mediated phagocytosis	Up-regulated	1.45	0.04

## Data Availability

The data presented in this study are available on request from the corresponding author.

## Supplementary Materials

The following supporting information can be downloaded at: https://www.mdpi.com/article/10.3390/ijms24129899/s1.
