# Supplementary material for: Proteomic Profiling of Chemotherapy Responses in FOLFOX-Resistant Colorectal Cancer Cells
Source: Int J Mol Sci. 2023 Jun 8;24(12):9899. doi: 10.3390/ijms24129899 (PMC10298463; doi:10.3390/ijms24129899)
Supplement: Supplementary file 1 [file ijms-24-09899-s001.zip › ijms-2428700-supplementary.pdf]

International Journal of *Molecular Sciences*

MDPI

Article

Proteomic Profiling of Chemotherapy Responses in FOLFOX-Resistant Colorectal Cancer Cells

Shing Yau Tam [1,*], Md Zahirul Islam Khan [1], Ju-Yu Chen [1], Jerica Hiu Yui Yip [1], Hong Yiu Yan [1], Tsz Yan Tam [1], Helen Ka Wai Law [1]

Table S1. List of DEPs in DLD1-R and DLD1 comparison

Protein (Gene symbol)	Up-regulated or Down-regulated	Log2 FC	p value	q value
NDUFAF1	Up-regulated	13.28771	0.000193	0.010875
GTPBP1	Up-regulated	13.28771	2.21E-06	0.000518
PSMG3	Up-regulated	13.28771	9.92E-08	0.000139
BRIX1	Up-regulated	13.28771	0.00041	0.01611
SNX27	Up-regulated	13.28771	1.77E-06	0.000505
BTAF1	Up-regulated	13.28771	1.08E-05	0.001682
UFC1	Up-regulated	13.28771	2.45E-07	0.000172
HINT3	Up-regulated	8.124222	0.027485	0.073622
PPP6R1	Up-regulated	6.376713	0.045393	0.093669
LEMD3	Up-regulated	5.84017	0.039771	0.086704
NOC3L	Up-regulated	5.489149	0.000659	0.021423
BRCC3	Up-regulated	5.260601	0.049814	0.09817
VPS53	Up-regulated	5.157159	0.005494	0.038633
KRT80	Up-regulated	4.896853	0.034429	0.081925
CNBP	Up-regulated	4.66366	0.002963	0.031266
LYRM7	Up-regulated	4.63208	0.005412	0.038633
MRPL19	Up-regulated	4.111114	0.00101	0.02548
BLMH	Up-regulated	4.025262	0.004822	0.037468
HMOX1	Up-regulated	3.636482	0.029916	0.076076
ARL6IP1	Up-regulated	3.550774	0.037998	0.085498
UBA5	Up-regulated	3.276298	0.002902	0.031152
OSBPL1A	Up-regulated	3.235296	0.001328	0.025747
SULT1A3	Up-regulated	3.208789	0.011568	0.050237
MRPS36	Up-regulated	3.207862	0.040524	0.087733
ACSL1	Up-regulated	3.186404	0.018233	0.059764
RAB5IF	Up-regulated	3.11439	0.009	0.0458
KPNA5	Up-regulated	2.953091	0.043919	0.091536
UNC119B	Up-regulated	2.869334	0.006406	0.04132
UBR5	Up-regulated	2.791099	0.007921	0.043686
EBNA1BP2	Up-regulated	2.661583	0.041532	0.088633
GMPR2	Up-regulated	2.631457	0.003518	0.032285
NUP98	Up-regulated	2.613562	0.010979	0.049249
PARM1	Up-regulated	2.442218	0.009159	0.0458
OXSR1	Up-regulated	2.43619	0.0228	0.066593
STUB1	Up-regulated	2.426633	0.017698	0.058787

PSAP	Up-regulated	2.388984	0.047765	0.09634
MLST8	Up-regulated	2.378344	0.022966	0.066593
MRFAP1	Up-regulated	2.334208	0.040933	0.087885
ITPA	Up-regulated	2.275218	0.001209	0.02548
MOV10	Up-regulated	2.225805	0.031307	0.077989
DKC1	Up-regulated	2.213916	0.022273	0.06585
TMX2	Up-regulated	2.137586	0.002076	0.028627
SPAG9	Up-regulated	2.135847	0.003122	0.031308
ALDH5A1	Up-regulated	2.126964	0.004811	0.037468
OGFOD1	Up-regulated	2.112502	0.000175	0.010875
PSMF1	Up-regulated	2.055049	0.02104	0.06406
MAVS	Up-regulated	2.028624	0.004311	0.034571
HSPB3	Up-regulated	2.003496	0.000874	0.024113
COMTD1	Up-regulated	1.968128	0.011716	0.050237
CPNE1	Up-regulated	1.967748	0.00431	0.034571
DPP7	Up-regulated	1.961765	0.005027	0.038418
TOM1L1	Up-regulated	1.952198	0.019363	0.061466
KRT20	Up-regulated	1.950988	0.006406	0.04132
DMAC1	Up-regulated	1.922818	0.00268	0.029914
ADGRG1	Up-regulated	1.913735	0.039592	0.086592
RRS1	Up-regulated	1.887555	0.006961	0.042006
SQSTM1	Up-regulated	1.864131	0.000354	0.015065
CSNK1A1	Up-regulated	1.784997	0.014225	0.053347
LRBA	Up-regulated	1.780563	0.002133	0.028816
MRPL15	Up-regulated	1.76193	0.001529	0.025747
STK26	Up-regulated	1.750187	0.022911	0.066593
C8orf82	Up-regulated	1.72459	0.028484	0.074846
BCAR1	Up-regulated	1.712498	0.001434	0.025747
TAX1BP1	Up-regulated	1.692341	0.04546	0.093669
MRPL52	Up-regulated	1.673384	0.025405	0.070343
SEC23B	Up-regulated	1.668719	0.006725	0.042006
SH3BGRL	Up-regulated	1.653075	0.040328	0.08752
DDX27	Up-regulated	1.652407	0.000686	0.021423
GNA11	Up-regulated	1.648165	0.001529	0.025747
PLXNB2	Up-regulated	1.642496	0.009127	0.0458
BAZ1B	Up-regulated	1.632471	0.005374	0.038633
SF3B5	Up-regulated	1.621415	0.006729	0.042006
ARFGAP1	Up-regulated	1.605696	0.011676	0.050237
EDF1	Up-regulated	1.6024	0.020124	0.062335
WDR55	Up-regulated	1.58698	0.040616	0.087733
ECI1	Up-regulated	1.572797	0.015272	0.054547
RPL30	Up-regulated	1.572349	5.24E-05	0.005669
DYNC1LI2	Up-regulated	1.56813	0.01977	0.061921
YTHDF3	Up-regulated	1.561097	1.01E-05	0.001682
MRPL47	Up-regulated	1.56076	0.000184	0.010875
ZC3H14	Up-regulated	1.554807	0.038718	0.086184
PPL	Up-regulated	1.551118	0.014876	0.054337
ITPR3	Up-regulated	1.5308	0.002563	0.029308

UBXN4	Up-regulated	1.530761	0.002948	0.031266
SLC38A10	Up-regulated	1.526209	0.03066	0.07727
CFAP298	Up-regulated	1.514938	0.006487	0.04132
UACA	Up-regulated	1.487945	0.046897	0.095444
FAM120A	Up-regulated	1.483391	0.009347	0.04596
MPI	Up-regulated	1.48335	0.041123	0.088157
DNMT1	Up-regulated	1.474454	4.58E-05	0.005369
DCAF1	Up-regulated	1.459656	0.012668	0.051341
GADD45GIP1	Up-regulated	1.449983	0.028925	0.075018
DIS3L2	Up-regulated	1.446853	0.035738	0.083022
MRPL44	Up-regulated	1.411095	0.00412	0.034282
H2AC17	Up-regulated	1.370916	0.047817	0.09634
H2AC8	Up-regulated	1.370916	0.047817	0.09634
EPS8L2	Up-regulated	1.368322	0.001915	0.027275
DHRS7	Up-regulated	1.36749	0.010916	0.049249
PWWP3A	Up-regulated	1.358746	0.003654	0.032338
DOK1	Up-regulated	1.355675	0.02011	0.062335
CUL2	Up-regulated	1.351338	0.002407	0.028871
MGST1	Up-regulated	1.344361	0.034953	0.082222
ASPSCR1	Up-regulated	1.330615	0.001969	0.027697
EVPL	Up-regulated	1.324235	0.002308	0.028816
LANCL2	Up-regulated	1.318905	0.014027	0.052986
YBX1	Up-regulated	1.304236	0.010218	0.048148
DDB1	Up-regulated	1.299446	0.001505	0.025747
NEDD8	Up-regulated	1.28025	0.001157	0.02548
GCC2	Up-regulated	1.270937	0.015266	0.054547
RPS17	Up-regulated	1.269232	0.000703	0.021453
GRSF1	Up-regulated	1.267074	0.001392	0.025747
CKAP5	Up-regulated	1.266739	0.007028	0.042006
RNF40	Up-regulated	1.266436	0.004098	0.034282
MRPL48	Up-regulated	1.265392	0.003467	0.032285
PNO1	Up-regulated	1.25633	0.033591	0.08075
TSR1	Up-regulated	1.253829	0.020174	0.062353
MRPL49	Up-regulated	1.252097	0.016049	0.055866
DYNC1I2	Up-regulated	1.228328	0.005884	0.039975
TOLLIP	Up-regulated	1.218308	0.02068	0.0635
XRCC1	Up-regulated	1.217497	0.046034	0.09398
IGF2BP3	Up-regulated	1.215942	0.01324	0.05182
GIPC1	Up-regulated	1.212122	0.026498	0.07182
MRPL9	Up-regulated	1.210731	0.044709	0.092872
FASTKD2	Up-regulated	1.203495	0.030506	0.077021
RPS23	Up-regulated	1.196371	0.048175	0.096645
MRPL54	Up-regulated	1.193378	0.007241	0.042429
IGF2R	Up-regulated	1.184064	0.001569	0.025747
IK	Up-regulated	1.174894	0.010469	0.048816
H1-4	Up-regulated	1.141335	0.022311	0.06585
RPS16	Up-regulated	1.139024	0.001127	0.02548
MRPS31	Up-regulated	1.118756	0.045492	0.093669

TIMM17B	Up-regulated	1.118467	0.006599	0.041803
MRPL12	Up-regulated	1.112012	0.000981	0.02548
NDUFAF3	Up-regulated	1.111368	0.009145	0.0458
CTSD	Up-regulated	1.106826	0.003001	0.031266
UBE2V1	Up-regulated	1.098154	0.006882	0.042006
GLIPR2	Up-regulated	1.086264	0.037507	0.085074
TRERF1	Up-regulated	1.082017	0.039285	0.086458
FBXO21	Up-regulated	1.079296	0.049253	0.097832
FAM91A1	Up-regulated	1.062798	0.015634	0.055037
SNX5	Up-regulated	1.061431	0.03316	0.080561
RRBP1	Up-regulated	1.060565	0.003081	0.031308
RPS25	Up-regulated	1.058254	0.012636	0.051341
SCO2	Up-regulated	1.042733	0.010612	0.049212
ELAC2	Up-regulated	1.041691	0.008179	0.044411
RWDD1	Up-regulated	1.040827	0.022096	0.065694
UAP1	Up-regulated	1.037616	0.014308	0.053374
TALDO1	Up-regulated	1.023369	0.001115	0.02548
PAK1	Up-regulated	1.022001	0.035058	0.082307
RPL14	Up-regulated	1.018311	0.016266	0.056067
RPS11	Up-regulated	1.016802	0.000211	0.011395
RPL8	Up-regulated	1.015712	0.002672	0.029914
SCLY	Up-regulated	1.002226	0.044779	0.092881
GSR	Down-regulated	-1.00473	0.040654	0.087733
UFD1	Down-regulated	-1.00502	0.01027	0.048148
ADK	Down-regulated	-1.009	0.001515	0.025747
HSP90AB1	Down-regulated	-1.01378	0.000342	0.015018
PREB	Down-regulated	-1.01819	0.013573	0.05222
TRMT10C	Down-regulated	-1.0206	0.021453	0.064562
GNPNAT1	Down-regulated	-1.02559	0.00229	0.028816
SSB	Down-regulated	-1.03028	0.029739	0.076076
RBM14	Down-regulated	-1.03928	0.029073	0.075018
HLA-B	Down-regulated	-1.04007	0.026556	0.07182
PTPN1	Down-regulated	-1.0448	0.005483	0.038633
NCEH1	Down-regulated	-1.04585	0.012293	0.050887
VCL	Down-regulated	-1.04621	6.24E-05	0.005853
COPG1	Down-regulated	-1.04695	0.017271	0.057829
POLR1C	Down-regulated	-1.04814	0.032927	0.080252
ZC3H18	Down-regulated	-1.04864	0.018745	0.060463
RRM1	Down-regulated	-1.06444	0.002802	0.030782
TPM3	Down-regulated	-1.07115	0.006818	0.042006
COPA	Down-regulated	-1.07324	0.000122	0.00961
PFDN2	Down-regulated	-1.07429	0.023057	0.066719
ZNF207	Down-regulated	-1.08663	0.005881	0.039975
CA2	Down-regulated	-1.09138	0.000125	0.00961
COTL1	Down-regulated	-1.09264	0.02131	0.06431
NUDCD1	Down-regulated	-1.10772	0.034955	0.082222
PVR	Down-regulated	-1.11021	0.032528	0.079973
CD44	Down-regulated	-1.11864	0.046997	0.095508

MAP2K2	Down-regulated	-1.12575	0.016168	0.056003
SFXN1	Down-regulated	-1.13649	0.024601	0.069331
IPO9	Down-regulated	-1.13657	0.024028	0.068263
XPO5	Down-regulated	-1.13915	0.029543	0.075954
DIAPH1	Down-regulated	-1.14247	0.010656	0.049212
MTA2	Down-regulated	-1.15572	0.003061	0.031308
SSBP1	Down-regulated	-1.1599	0.039137	0.086266
EMC8	Down-regulated	-1.16088	0.049163	0.09779
CFL2	Down-regulated	-1.16342	0.007552	0.043196
NUDT19	Down-regulated	-1.17045	0.028785	0.074964
NDUFS6	Down-regulated	-1.21056	0.003412	0.032285
ALG5	Down-regulated	-1.21056	0.008289	0.044516
UBA6	Down-regulated	-1.21122	0.013592	0.05222
MT-CO1	Down-regulated	-1.21646	0.006853	0.042006
PPP2R1B	Down-regulated	-1.22537	0.041669	0.088735
PLOD1	Down-regulated	-1.22671	0.012852	0.051415
AASDHPPT	Down-regulated	-1.23227	0.011882	0.050325
VPS26B	Down-regulated	-1.23856	0.005373	0.038633
CXCL5	Down-regulated	-1.25777	0.007728	0.043298
HAT1	Down-regulated	-1.26226	0.032237	0.079536
CAMSAP3	Down-regulated	-1.26303	0.023542	0.067429
CDV3	Down-regulated	-1.26378	0.008095	0.044347
ZNF221	Down-regulated	-1.26573	0.015502	0.055037
NLRP2	Down-regulated	-1.26727	0.02763	0.073732
AK4	Down-regulated	-1.26929	0.001664	0.026598
HMGB2	Down-regulated	-1.27027	0.008711	0.045126
VDAC3	Down-regulated	-1.28179	0.007776	0.043392
HMOX2	Down-regulated	-1.28205	0.000412	0.01611
PPAT	Down-regulated	-1.28431	0.002827	0.03082
CHAC2	Down-regulated	-1.29388	0.000364	0.015071
KHSRP	Down-regulated	-1.29629	0.015654	0.055037
KRT13	Down-regulated	-1.30795	0.005486	0.038633
PPP4C	Down-regulated	-1.30803	0.036549	0.083663
TPM1	Down-regulated	-1.31387	0.019835	0.061985
DDAH1	Down-regulated	-1.32861	0.039125	0.086266
PNP	Down-regulated	-1.3298	0.00029	0.013599
CSK	Down-regulated	-1.34083	0.006493	0.04132
CIAPIN1	Down-regulated	-1.34382	0.013172	0.05182
PPP1CA	Down-regulated	-1.34452	0.001741	0.026908
SETDB1	Down-regulated	-1.34639	0.005732	0.039628
PHGDH	Down-regulated	-1.35245	0.031685	0.078674
NDUFB9	Down-regulated	-1.37445	0.014928	0.054387
ACLY	Down-regulated	-1.37874	5.67E-05	0.005693
GCN1	Down-regulated	-1.37935	0.014775	0.054251
MCM5	Down-regulated	-1.39063	0.049611	0.098126
TXNDC5	Down-regulated	-1.39804	0.035509	0.082952
VCP	Down-regulated	-1.4006	0.022573	0.066272
P4HA1	Down-regulated	-1.40932	0.012553	0.051341

DNAJB1	Down-regulated	-1.41079	0.013339	0.05182
DDX3X	Down-regulated	-1.46054	0.01161	0.050237
CRIP2	Down-regulated	-1.4814	0.000542	0.019531
TTC9C	Down-regulated	-1.48393	0.045276	0.093635
IGF2BP2	Down-regulated	-1.49627	0.016471	0.056496
PPP1CB	Down-regulated	-1.49758	0.001027	0.02548
HEBP1	Down-regulated	-1.52216	0.024902	0.069623
ZRANB2	Down-regulated	-1.54801	0.00225	0.028816
ACBD3	Down-regulated	-1.55192	0.00367	0.032338
NUP54	Down-regulated	-1.57685	0.00189	0.027275
CDCP1	Down-regulated	-1.58118	0.004214	0.034571
EEF1A2	Down-regulated	-1.58782	0.000132	0.00961
PARP1	Down-regulated	-1.61294	0.000684	0.021423
GPRC5A	Down-regulated	-1.61376	0.023969	0.068233
SARS1	Down-regulated	-1.64637	0.000427	0.016234
PPFIBP1	Down-regulated	-1.68769	0.015048	0.054517
TXNL1	Down-regulated	-1.6898	0.002315	0.028816
SCD	Down-regulated	-1.72556	0.039554	0.086592
P4HA2	Down-regulated	-1.74014	0.036238	0.083407
USP14	Down-regulated	-1.75496	0.00635	0.04132
SNAPIN	Down-regulated	-1.76094	0.005254	0.038633
GEMIN5	Down-regulated	-1.76169	0.00125	0.02548
ARHGEF1	Down-regulated	-1.77689	0.010767	0.049249
BAG3	Down-regulated	-1.77764	0.000225	0.011712
ICAM1	Down-regulated	-1.77848	0.043727	0.091509
EXOSC4	Down-regulated	-1.78567	0.019894	0.062032
SV2C	Down-regulated	-1.788	0.029908	0.076076
FSIP2	Down-regulated	-1.79383	0.003527	0.032285
PAPSS2	Down-regulated	-1.79676	0.010945	0.049249
FLYWCH2	Down-regulated	-1.80937	0.00939	0.046009
TCEA1	Down-regulated	-1.81742	0.011337	0.04998
PPP1R2	Down-regulated	-1.8266	0.005454	0.038633
TUBA4A	Down-regulated	-1.8401	0.000833	0.023768
MAPT	Down-regulated	-1.84122	0.030046	0.07617
KIF23	Down-regulated	-1.84491	0.00749	0.043196
BRI3BP	Down-regulated	-1.85383	0.006044	0.040668
CCDC51	Down-regulated	-1.86404	0.012303	0.050887
RBM10	Down-regulated	-1.88103	0.015158	0.054517
COPS5	Down-regulated	-1.97703	0.014569	0.053899
RFC2	Down-regulated	-1.97958	0.036389	0.083617
RAB11FIP1	Down-regulated	-1.99416	0.009819	0.046966
RNASEH2B	Down-regulated	-2.02213	0.002163	0.028816
RUNDC3B	Down-regulated	-2.05574	0.032668	0.079973
WDR12	Down-regulated	-2.05943	0.016981	0.057765
CPSF7	Down-regulated	-2.06083	0.028877	0.075018
CNN3	Down-regulated	-2.07011	0.009553	0.046328
METTL26	Down-regulated	-2.13054	0.010248	0.048148
FAM241A	Down-regulated	-2.14875	0.002315	0.028816

RALA	Down-regulated	-2.15367	0.011069	0.049416
MTX2	Down-regulated	-2.17174	0.016586	0.056752
GNG12	Down-regulated	-2.18123	0.018935	0.060794
LUC7L	Down-regulated	-2.18515	0.006963	0.042006
IL18	Down-regulated	-2.23594	0.003865	0.033344
ASMTL	Down-regulated	-2.25416	0.039489	0.086592
KIF11	Down-regulated	-2.27129	0.009093	0.0458
HMGA1	Down-regulated	-2.31195	0.019135	0.061288
GNAQ	Down-regulated	-2.39066	0.022347	0.06585
EIF4EBP2	Down-regulated	-2.41947	0.030715	0.07727
SMNDC1	Down-regulated	-2.44838	0.010673	0.049212
RECQL	Down-regulated	-2.45061	0.000137	0.00961
RRM2	Down-regulated	-2.5097	0.002176	0.028816
GNAS	Down-regulated	-2.51788	0.002464	0.028871
CCDC124	Down-regulated	-2.5724	0.001785	0.027275
PFDN4	Down-regulated	-2.58141	0.00876	0.045126
GOLGA5	Down-regulated	-2.6136	0.014596	0.053899
LCN2	Down-regulated	-2.62505	0.004599	0.036333
ZNF598	Down-regulated	-2.87025	0.003098	0.031308
HPRT1	Down-regulated	-2.87626	0.000682	0.021423
PBK	Down-regulated	-2.95987	0.013399	0.051907
EHD2	Down-regulated	-2.97117	0.008178	0.044411
NDUFB3	Down-regulated	-3.00475	0.034694	0.082137
FBH1	Down-regulated	-3.07515	0.00389	0.033353
EXOC7	Down-regulated	-3.1304	0.003326	0.032285
ZPR1	Down-regulated	-3.25085	0.001112	0.02548
EML4	Down-regulated	-3.29131	0.001683	0.026598
CADM1	Down-regulated	-3.49778	0.003818	0.033141
MYO1E	Down-regulated	-3.63692	0.014603	0.053899
MRPL39	Down-regulated	-3.71186	0.010885	0.049249
AP2A2	Down-regulated	-6.39318	0.038514	0.086184
NDUFA6	Down-regulated	-6.76389	0.033722	0.080926
MAPRE2	Down-regulated	-13.2877	4.79E-07	0.000225
SPARC	Down-regulated	-13.2877	5.17E-06	0.001039

Table S2. List of DEPs in HCT116-R and HCT116 comparison

Protein (Gene symbol)	Up-regulated or Down-regulated	Log2 FC	p value	q value
SPARC	Up-regulated	13.28771	0.0008088	0.086075
HSPB3	Up-regulated	4.232121	0.0128787	0.213207
KRT7	Up-regulated	3.956493	0.0037939	0.144266
MAL2	Up-regulated	3.588477	0.0054075	0.154885
SLC1A3	Up-regulated	3.529859	0.0384931	0.277843
APOBEC3G	Up-regulated	3.390062	2.89E-05	0.039531
NCAM1	Up-regulated	3.320068	0.0045049	0.147131
DCUN1D3	Up-regulated	2.503125	0.000116	0.039531
PCDH1	Up-regulated	2.274732	0.0211774	0.241679
PPIL1	Up-regulated	2.137416	0.0154127	0.223155
IL18	Up-regulated	2.100085	0.0002295	0.046007
DHRS1	Up-regulated	2.080868	0.0168185	0.223155
APOBEC3F	Up-regulated	2.027802	0.0344024	0.265474
RPEL1	Up-regulated	2.008542	0.0291546	0.25293
S100A4	Up-regulated	1.926389	0.0001314	0.039531
ALDH2	Up-regulated	1.912596	0.0250027	0.246468
PPFIBP1	Up-regulated	1.837671	0.0011696	0.095931
LGALS3BP	Up-regulated	1.827325	7.91E-05	0.039531
EFHD2	Up-regulated	1.798583	0.003454	0.144266
GMPPB	Up-regulated	1.753767	0.0388842	0.278214
SH3KBP1	Up-regulated	1.733082	0.0134312	0.216694
RDH14	Up-regulated	1.71833	0.0278807	0.249063
HAGH	Up-regulated	1.605864	0.0073498	0.178747
D99	Up-regulated	1.583272	0.0091407	0.190682
IFITM1	Up-regulated	1.578889	0.0119274	0.208381
ACSF2	Up-regulated	1.505225	0.0053325	0.154885
EPB41L1	Up-regulated	1.466588	0.00167	0.115902
DUSP3	Up-regulated	1.445444	0.0455231	0.28608
ASMTL	Up-regulated	1.444255	0.0146215	0.223155
TMED5	Up-regulated	1.434339	0.041331	0.281238
FLNB	Up-regulated	1.339116	0.000869	0.086075
HMGN5	Up-regulated	1.306786	0.0087141	0.190682
PTPN11	Up-regulated	1.271055	0.0028237	0.137711
PODXL	Up-regulated	1.231556	0.0228406	0.245332
EPHA2	Up-regulated	1.213149	0.0137485	0.217552
MACF1	Up-regulated	1.190548	0.0370292	0.274317
HTATIP2	Up-regulated	1.179904	0.0049341	0.151134
AK4	Up-regulated	1.172502	0.0021836	0.121026
ATP6V1G1	Up-regulated	1.162766	0.0166058	0.223155
HGS	Up-regulated	1.122885	0.0329003	0.262693
PACSIN2	Up-regulated	1.114835	0.0213292	0.241679
MLEC	Up-regulated	1.0869	0.007494	0.178747
PPP1R18	Up-regulated	1.085596	0.0230449	0.246062

MYOF	Up-regulated	1.083189	0.0001283	0.039531
ALDH5A1	Up-regulated	1.081955	0.0452481	0.28608
S100A11	Up-regulated	1.075463	0.0131942	0.216445
FAM169A	Up-regulated	1.059988	0.0414572	0.281238
ADAM10	Up-regulated	1.024902	0.0117521	0.208381
CD2AP	Up-regulated	1.012843	0.0153793	0.223155
FAM120A	Down-regulated	-1.01068	0.0487773	0.290898
COPS7A	Down-regulated	-1.02697	0.0164134	0.223155
MAOA	Down-regulated	-1.02961	0.0479846	0.290898
CDC37L1	Down-regulated	-1.04342	0.0226985	0.245332
RNF20	Down-regulated	-1.04798	0.0466887	0.289191
SRSF7	Down-regulated	-1.05522	0.0213796	0.241679
NNT	Down-regulated	-1.05553	0.0161629	0.223155
TBK1	Down-regulated	-1.09051	0.013985	0.217552
PYCR2	Down-regulated	-1.13918	0.018829	0.231135
GTF2E1	Down-regulated	-1.14726	0.0357689	0.269771
RAP1GDS1	Down-regulated	-1.15832	0.0057532	0.159716
DCAF7	Down-regulated	-1.15991	0.0253812	0.246468
MSH6	Down-regulated	-1.1641	0.0470057	0.289191
LAMB1	Down-regulated	-1.17781	0.0375546	0.275477
SLK	Down-regulated	-1.27133	0.0120977	0.208381
FKBP8	Down-regulated	-1.30087	0.0271783	0.248361
TRGC1	Down-regulated	-1.3066	0.02151	0.241679
TCF7L2	Down-regulated	-1.3624	0.0285155	0.250344
UTP18	Down-regulated	-1.43594	0.0162928	0.223155
FHAD1	Down-regulated	-1.44845	0.0082325	0.190682
PCID2	Down-regulated	-1.47524	0.0433648	0.28608
MAOB	Down-regulated	-1.47728	0.025171	0.246468
NABP2	Down-regulated	-1.56225	0.0296994	0.253672
OR51L1	Down-regulated	-1.57975	0.008846	0.190682
MTA1	Down-regulated	-1.61299	0.0263538	0.247685
EIF2D	Down-regulated	-1.62638	0.0267024	0.248361
CDKAL1	Down-regulated	-1.64445	0.0499353	0.294471
AKAP12	Down-regulated	-1.65645	0.0014755	0.110936
ZAP70	Down-regulated	-1.68626	0.016048	0.223155
TSC22D1	Down-regulated	-1.76922	0.0085793	0.190682
SCLY	Down-regulated	-1.8362	0.0030465	0.143149
ZKSCAN5	Down-regulated	-1.88322	0.0325283	0.262041
EEF1AKMT2	Down-regulated	-1.88958	0.0069692	0.175691
JAG1	Down-regulated	-2.15852	0.0040918	0.144778
TIMM50	Down-regulated	-2.30383	0.0242443	0.246468
SNAPIN	Down-regulated	-2.34203	0.0073243	0.178747
PALLD	Down-regulated	-3.15223	5.48E-05	0.039531
BIRC6	Down-regulated	-3.30747	0.0434749	0.28608
TGM3	Down-regulated	-3.33022	0.0381725	0.277843
CADM1	Down-regulated	-3.3899	0.0334706	0.264667
CRIP1	Down-regulated	-6.16366	0.0005197	0.072135

Table S3. GSEA details of the up-regulation of ribosome in DLD1-R

Protein (Gene symbol)	Rank in Gene List	Rank Metric Score	Running Enrichment Score	Core Enrichment
RPL30	23	2.484	0.0291	Yes
RPS17	55	2.068	0.0488	Yes
RPS16	68	1.877	0.0728	Yes
RPS11	91	1.692	0.0902	Yes
RPL8	92	1.691	0.1159	Yes
RPL14	101	1.622	0.1375	Yes
RPS9	103	1.615	0.1616	Yes
RPL32	112	1.577	0.1826	Yes
RPS25	119	1.559	0.204	Yes
RPS2	122	1.53	0.2264	Yes
RPS3A	140	1.471	0.2424	Yes
RPL18	141	1.469	0.2647	Yes
RPL5	142	1.466	0.2869	Yes
RPL17	144	1.46	0.3087	Yes
RPS23	156	1.419	0.3261	Yes
RPS10	168	1.376	0.3428	Yes
RPL11	179	1.34	0.3594	Yes
RPS7	184	1.326	0.378	Yes
RPS20	188	1.295	0.3966	Yes
RPL10	193	1.275	0.4144	Yes
RPL19	194	1.274	0.4337	Yes
RPS28	206	1.228	0.4483	Yes
RPL13	207	1.225	0.4669	Yes
RPS21	211	1.213	0.4841	Yes
RPS24	236	1.154	0.4926	Yes
RPS3	240	1.147	0.5089	Yes
RPS5	257	1.114	0.5198	Yes
RPL23	273	1.063	0.5303	Yes
RPL38	276	1.058	0.5456	Yes
RPS8	290	1.028	0.5564	Yes
RPSA	313	0.985	0.5631	Yes
RPL37A	318	0.972	0.5763	Yes
RPL6	350	0.914	0.5785	Yes
RPL9	362	0.902	0.5881	Yes
RPL3	396	0.853	0.5887	Yes
RPL27A	398	0.853	0.6013	Yes
RPL10A	405	0.834	0.6116	Yes
RPS13	442	0.792	0.6102	Yes
RPS15	457	0.777	0.6167	Yes
RPS19	458	0.775	0.6285	Yes
RPS12	459	0.772	0.6402	Yes

RPS18	529	0.683	0.6247	Yes
MRPL13	531	0.68	0.6346	Yes
RPS27L	532	0.678	0.6449	Yes
RPL22	534	0.674	0.6548	Yes
RPS15A	553	0.654	0.6579	Yes
RPL21	565	0.643	0.6636	Yes
RPL35A	567	0.64	0.6729	Yes
RPL18A	630	0.57	0.6583	Yes
RPL26L1	671	0.518	0.6512	Yes
RPS26	675	0.514	0.6579	Yes
FAU	692	0.499	0.6594	Yes
RPL35	699	0.494	0.6647	Yes
RPL7	712	0.48	0.6675	Yes
RPL24	717	0.475	0.6732	Yes
RPL12	814	0.37	0.6428	No
RPS27	830	0.359	0.6426	No
RPL7A	946	0.262	0.6035	No
RPL27	963	0.241	0.6012	No
RPL36	1107	0.138	0.5497	No
RPS4X	1131	0.113	0.5428	No
RPS6	1184	0.069	0.5243	No
RPL15	1215	0.051	0.5138	No
RPL23A	1239	0.03	0.5057	No
RPL22L1	1414	-0.1	0.442	No
RPL4	1456	-0.138	0.4287	No
RPL29	1509	-0.173	0.4118	No
RPLP0	1631	-0.265	0.3705	No
RPS27A	1636	-0.268	0.3731	No
RPLP2	2051	-0.583	0.2267	No
RPLP1	2301	-0.818	0.1458	No
RPL13A	2523	-1.185	0.081	No

Table S4. GSEA details of the down-regulation of DNA replication in DLD1-R

Protein (Gene symbol)	Rank in Gene List	Rank Metric Score	Running Enrichment Score	Core Enrichment
LIG1	640	0.56	-0.1991	No
MCM7	863	0.335	-0.2591	No
MCM6	1266	0.014	-0.4059	No
MCM3	1289	-0.006	-0.4136	No
RPA2	1937	-0.492	-0.6197	No
FEN1	2130	-0.652	-0.6482	Yes
MCM2	2136	-0.658	-0.6076	Yes
RFC3	2154	-0.673	-0.5704	Yes
PCNA	2196	-0.707	-0.5399	Yes
RFC5	2202	-0.712	-0.4958	Yes
RPA1	2206	-0.719	-0.4506	Yes
RFC4	2225	-0.739	-0.4096	Yes
RPA3	2230	-0.751	-0.3626	Yes
MCM4	2418	-0.978	-0.3683	Yes
MCM5	2427	-0.992	-0.3072	Yes
SSBP1	2488	-1.099	-0.2584	Yes
POLE3	2546	-1.263	-0.1979	Yes
RFC2	2613	-1.491	-0.126	Yes
RNASEH2B	2725	-2.666	0.0051	Yes

Table S5. GSEA details of the up-regulation of actin cytoskeleton in HCT116-R

Protein (Gene symbol)	Rank in Gene List	Rank Metric Score	Running Enrichment Score	Core Enrichment
KRAS	32	1.383	0.0371	Yes
ROCK2	36	1.352	0.0839	Yes
EZR	41	1.334	0.1297	Yes
MYH14	56	1.241	0.1684	Yes
ARPC3	89	1.095	0.1953	Yes
ARPC5	106	1.003	0.2249	Yes
MSN	109	0.994	0.2594	Yes
ARPC5L	131	0.894	0.2832	Yes
PXN	150	0.847	0.3065	Yes
MYL12B	244	0.716	0.2972	Yes
RRAS2	255	0.698	0.3182	Yes
BAIAP2	262	0.689	0.3404	Yes
PFN2	319	0.618	0.3413	Yes
ACTN4	325	0.612	0.3612	Yes
SSH3	335	0.604	0.3792	Yes
RHOA	408	0.552	0.3719	Yes
NRAS	443	0.532	0.3781	Yes
DIAPH1	474	0.51	0.3849	Yes
MYH9	521	0.482	0.3848	Yes
VCL	544	0.47	0.3933	Yes
ARPC2	564	0.458	0.4024	Yes
BCAR1	571	0.453	0.4162	Yes
ACTN1	599	0.439	0.4217	Yes
CDC42	633	0.421	0.4243	Yes
CRK	680	0.395	0.4211	Yes
ARHGEF1	701	0.389	0.4275	Yes
PAK1	746	0.359	0.4238	Yes
ARPC1B	797	0.335	0.4169	Yes
PPP1CA	808	0.328	0.4248	Yes
ITGA6	843	0.313	0.4232	Yes
PIP4K2C	845	0.313	0.434	Yes
CFL2	867	0.304	0.4369	Yes
PPP1R12A	873	0.302	0.4457	Yes
PPP1CB	898	0.285	0.4469	Yes
TMSB4X	979	0.249	0.4258	No
PFN1	1001	0.24	0.4265	No
MYH10	1012	0.237	0.4311	No
ARPC4	1173	0.17	0.3774	No
IQGAP1	1220	0.156	0.3657	No
RAC1	1245	0.147	0.362	No
MAP2K1	1314	0.12	0.3408	No

CYFIP2	1408	0.084	0.3091	No
NCKAP1	1415	0.082	0.3098	No
CFL1	1416	0.082	0.3127	No
GIT1	1429	0.075	0.3109	No
MAP2K2	1444	0.068	0.308	No
BRAF	1500	0.041	0.2889	No
GSN	1551	0.016	0.2708	No
ITGAV	1557	0.012	0.2694	No
GNG12	1670	-0.032	0.2287	No
ARPC1A	1671	-0.033	0.2299	No
RDX	1693	-0.04	0.2234	No
ENAH	1756	-0.064	0.2025	No
ACTB	1877	-0.122	0.162	No
CYFIP1	1900	-0.136	0.1586	No
ITGA2	1910	-0.142	0.1603	No
ITGB4	1913	-0.144	0.1647	No
ROCK1	1990	-0.183	0.1428	No
PIP4K2A	2020	-0.198	0.1389	No
ITGA3	2064	-0.223	0.1308	No
PAK2	2136	-0.258	0.1134	No
CRKL	2165	-0.276	0.1127	No
ITGB1	2254	-0.33	0.0916	No
CSK	2388	-0.451	0.0579	No
MAPK1	2464	-0.537	0.0489	No
PAK4	2554	-0.686	0.04	No
EGFR	2629	-0.854	0.0426	No

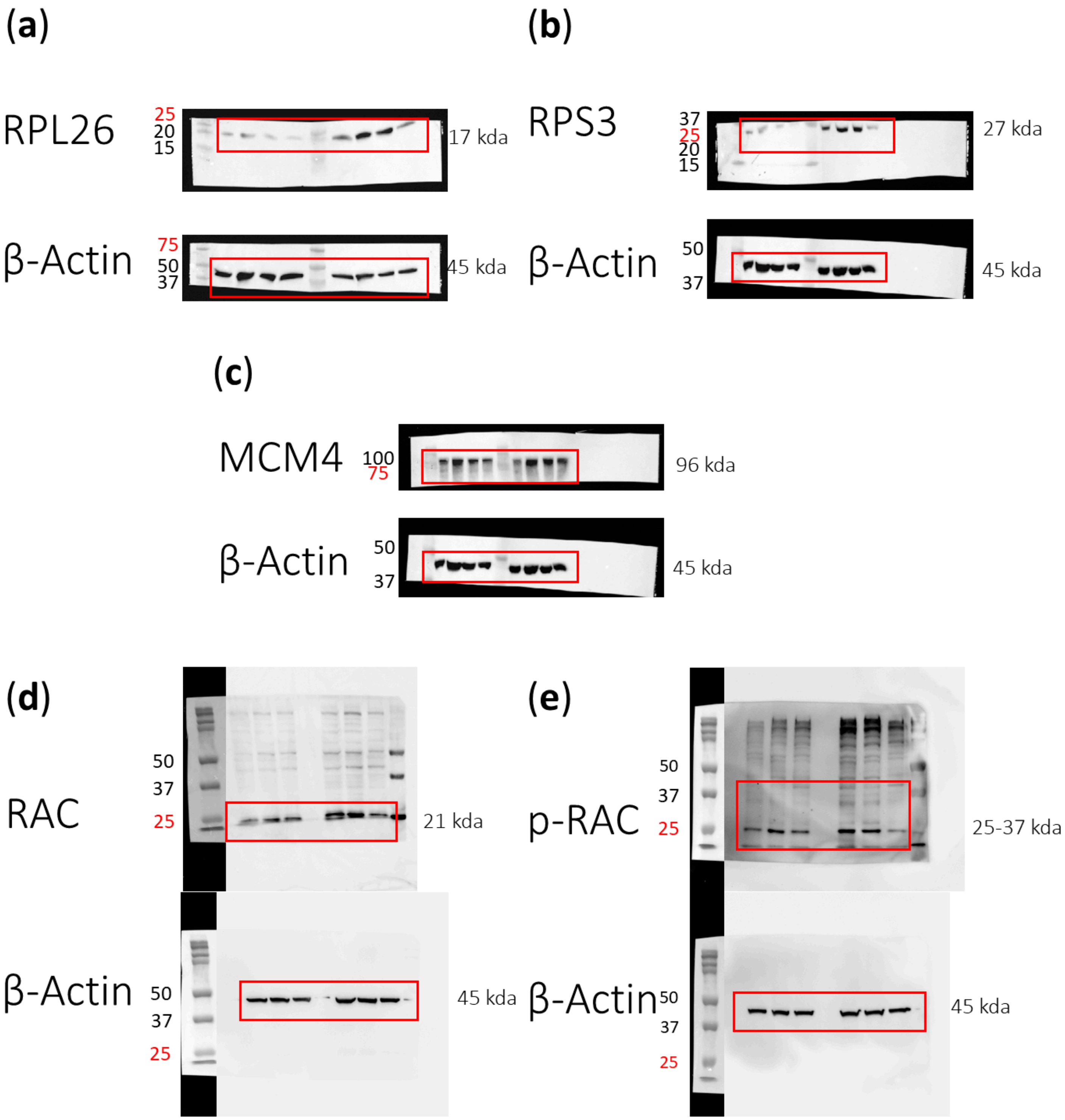

Figure S1. Original western blots of Figure 6
